# 2-Bromo-1,4-Naphthalenedione promotes CD8^+^ T cell expansion and limits Th1/Th17 to mitigate experimental autoimmune encephalomyelitis

**DOI:** 10.1186/s12974-024-03172-x

**Published:** 2024-07-27

**Authors:** Cuixia Yang, Yuanchen Ma, Qiying Lu, Yuliang Qu, Yuantao Li, Shimei Cheng, Chongjun Xiao, Jinshuo Chen, Chuangjia Wang, Feng Wang, Andy Peng Xiang, Weijun Huang, Xiaorong Tang, Haiqing Zheng

**Affiliations:** 1 Central Laboratory, Chaozhou Central Hospital Affiliated to Southern Medical University, Chaozhou, Guangdong Province China; 2 Department of Gastrointestinal Surgery, The First Affiliated Hospital, Sun Yat-sen University, Guangzhou, China; 3 Department of Rehabilitation Medicine, The Third Affiliated Hospital, Sun Yat-Sen University, No. 600 Tianhe Road, Guangzhou, China; 4 Key Laboratory for Stem Cells and Tissue Engineering, Sun Yat-sen University, Ministry of Education, Guangzhou, China; 5 Center for Stem Cell Biology and Tissue Engineering, Key Laboratory for Stem Cells and Tissue Engineering, Sun Yat-Sen University, Ministry of Education, Guangzhou, China; 6 Department of Biochemistry, Zhongshan School of Medicine, Sun Yat-Sen University, Guangzhou, China; 7 Shanghai Institute of Immunology, Shanghai Jiao Tong University School of Medicine, Shanghai, China

**Keywords:** 2-bromo-1,4-Naphthalenedione, CD8^+^ T cells, Experimental autoimmune encephalomyelitis, Th1, Th17

## Abstract

Treating Multiple sclerosis (MS), a well-known immune-mediated disease characterized by axonal demyelination, is challenging due to its complex causes. Naphthalenedione, present in numerous plants, is being explored as a potential medicine for MS due to its immunomodulatory properties. However, its effects on lymphocytes can vary depending on factors such as the specific compound, concentration, and experimental conditions. In this study, we aim to explore the therapeutic potential of 2-bromo-1,4-naphthalenedione (BrQ), a derivative of naphthalenedione, in experimental autoimmune encephalomyelitis (EAE), an animal model of MS, and to elucidate its underlying mechanisms. We observed that mice treated with BrQ exhibited reduced severity of EAE symptoms, including lower clinical scores, decreased leukocyte infiltration, and less extensive demyelination in central nervous system. Furthermore, it was noted that BrQ does not directly affect the remyelination process. Through cell-chat analysis based on bulk RNA-seq data, coupled with validation of flow analysis, we discovered that BrQ significantly promotes the expansion of CD8^+^ T cells and their interactions with other immune cells in peripheral immune system in EAE mice. Subsequent CD8^+^ T cell depletion experiments confirmed that BrQ alleviates EAE in a CD8^+^ T cell-dependent manner. Mechanistically, expanded CD8^+^ cells were found to selectively reduce antigen-specific CD4^+^ cells and subsequently inhibit Th1 and Th17 cell development in vivo, ultimately leading to relief from EAE. In summary, our findings highlight the crucial role of BrQ in modulating the pathogenesis of MS, suggesting its potential as a novel drug candidate for treating MS and other autoimmune diseases.

**Supplementary Information:**

The online version contains supplementary material available at 10.1186/s12974-024-03172-x.

## Introduction

Multiple sclerosis (MS) is the predominant chronic autoimmune disorder that affects the central nervous system (CNS), with a global impact on affecting nearly 3 million individuals. Typically appearing between the ages of 20 and 40, MS is the primary nontraumatic cause of neurologic disability among young adults. It disproportionately affects women, with a ratio of approximately two to one [1, 2]. Common symptoms of MS include limb weakness or sensory loss, ataxia, visual disturbances, spasticity, fatigue, pain, and cognitive deficits. Widely recognized today, MS is believed to stem from a complex interplay of multiple genetic, epigenetic, and environmental risk factors. Its pathogenesis unfolds through a series of pathological processes, beginning with immune system activation, followed by the infiltration of immune cells across the blood–brain barrier (BBB), demyelination, gliosis, and neuroaxonal degeneration. These cascading events culminate in the disruption of neuronal signaling, giving rise to neurologic syndromes and physical disability [3, 4].

Presently, clinical interventions primarily target the peripheral inflammatory processes that initiate during the early stages of MS. This treatment spectrum includes a variety of medications such as beta-interferons, teriflunomide, glatiramer acetate, dimethyl fumarate, mitoxantrone, ocrelizumab, and alemtuzumab. Recognizing the characteristic immune dysregulation observed in MS, which prompts the infiltration of autoreactive immune cells into the CNS and precipitates neurodegeneration, supplementary medications like fingolimod and natalizumab have been developed to further curb immune cell infiltration [5–7]. Although these therapies have demonstrated efficacy in alleviating disease severity, they only partially impede MS progression, falling short of achieving full remission. Furthermore, due to the lack of specificity in current treatments, the more potent medications for MS often entail a heightened risk of serious adverse events. For instance, adverse events associated with oral fingolimod in relapsing multiple sclerosis include bradycardia, atrioventricular conduction block, elevated liver enzyme levels, macular edema, and mild hypertension [8]. Similarly, natalizumab, along with other immunotherapeutic drugs, is linked to progressive multifocal leukoencephalopathy [7, 9]. Consequently, the therapeutic landscape for multiple sclerosis remains daunting, emphasizing the urgent need for the development of new medications with promising efficacy and fewer side effects.

For centuries, Traditional Chinese Medicines have been utilized in China and Europe to address a variety of illnesses. Fascinatingly, many of these medicines contain quinones, which possess diverse biological functions, including combating viruses, bacteria, fungi, malaria, cancer, and Alzheimer’s disease [10, 11]. Naphthoquinones, a subtype of quinone, have garnered significant attention in immunology and related fields. Research has demonstrated that 1,4-Naphthoquinone targets P2 × 7 purinergic receptors for anti-inflammatory effects [12, 13]. Lapachol reduces lymphocyte proliferation and severity of autoimmune arthritis [14]. Juglone, from walnut husks, has anti-diabetic, anti-hypertensive, anti-tumor properties, and improves ulcerative colitis [15]. Plumbagin, derived from medicinal plants, has been shown to ameliorate experimental autoimmune encephalomyelitis [16]. Shikonin in Lithospermum erythrorhizon is effective against various autoimmune diseases [17]. Vitamin K3 (menaquinone) may aid in the treatment of NLRP3-related inflammatory conditions [18]. These findings underscore the immunosuppressive effects of naphthoquinones, although their effects on lymphocytes can vary depending on factors such as the specific compound, concentration, and experimental conditions.

The experimental autoimmune encephalomyelitis (EAE) mouse model, which closely mimics many clinical, pathological, and histological features of MS, is widely utilized in MS drug discovery. We assessed the effects of 1,2-naphthoquinone, 1,4-naphthoquinone, 2-naphthol, menadione, 2,3-dichloro-1,4-naphthoquinone, quinoclamine, 2-chloro-1,4-naphthoquinone, 2-bromo-1,4-naphthoquinone, and 2-methoxy-1,4-naphthoquinone on EAE. The results demonstrated that BrQ exhibited the most significant and safe effect in alleviating EAE clinical symptoms, making it the most promising candidate for further investigation in MS treatment. In this study, the clinical scores, H&E and Luxol fast blue staining were used to determine the protective effect of BrQ on EAE mice. Oligodendrocyte precursor cells (OPCs) differentiation assay and cuprizone induced demyelination model were performed to evaluate the direct effect of BrQ on remyelination processes in vitro and in vivo, respectively. Further bulk RNA-seq, cell-chat analysis and flow analysis validation were conducted to uncover intercellular communication networks, as well as potential mechanisms and therapeutic targets. Overall, we show for the first time that BrQ significantly alleviates the severity of EAE mice and could be developed as a unique therapeutic agent for the treatment of MS and other autoimmune diseases.

## Materials and methods

### Mice

C57BL/6 mice were purchased from Beijing Vital River Laboratory Animal Technology (Beijing, China). All mice were maintained in pathogen-free conditions with standard laboratory chow and water ad libitum in the Sun Yat-sen University animal care facility. Sex- and age-matched littermates between 8 and 10 weeks of age were used for animal experiments. All animal experiments were performed with the approval of Sun Yat-sen University Institutional Animal Care and Use Committee.

### Induction and treatment of EAE

The EAE animal model was developed as previously described [19]. In brief, male C57BL/6 mice were immunized subcutaneously with 200 µg MOG_35 − 55_ peptide (MEVGWYRSPFSRVVHLYRNGK, GL Biochem, Shanghai, China) emulsified in Complete Freund’s adjuvant, which contains 5 mg/ml heat-killed Mycobacterium tuberculosis (H37Ra strain, BD Diagnostics). Each mouse received a total volume of 200 µl of MOG_35 − 55_ peptide/CFA emulsion, with 50 µl injected subcutaneously at two separate sites on the back, resulting in four injections per mouse. Pertussis toxin (dissolved in sterile distilled water, 200 ng/mouse, List Biological Labs) was injected intraperitoneally on day 0 and day 2 following immunization.

The mice were monitored daily and clinical appearance was scored on a scale of 0–5 as follows: 0, no clinical signs; 1, paralyzed tail; 2, paresis (weakness, incomplete paralysis of one or two hindlimbs); 3, totally paralyzed hind legs; 4, paraplegia with weak or paralyzed forelimbs; 5, death. Add or subtract 0.5 points if the disease severity is between two scales.

In the prophylactic treatment regimen, EAE mice were divided into four groups: vehicle, BrQ low dose group (3 mg/kg), BrQ medium dose group (10 mg/kg) and BrQ high dose group (30 mg/kg). 2-bromo-1,4-naphthalenedione (abbreviated as BrQ, Sigma, CAS: 2065-37-4, 510,300) was dissolved by sonication in a 0.5% carboxymethylcellulose sodium solution (CMC) suspension and aliquoted for freezing at -20 °C, with one vial used per extraction without repeated freeze-thaw cycles. The mice were orally administered at 9:00 am every day from day 3 post-immunization until the end of the study. The vehicle group was given an equal volume of 0.5% CMC (200 µL per mouse) by gavage. For the treatment regimen, the EAE mice were orally gavaged at a dosage of 30 mg/kg daily starting on day 7, day 14, or day 21 after immunization until the end of the study.

### Histopathological and immunohistochemical analysis

For the histological analysis, vehicle and BrQ-treated EAE mice were deeply anesthetized with 1.25% tribromoethanol (15 µL/g body weight) on day 18 post-immunization and then transcardially perfused with 20 ml 0.9% NaCl solution to remove peripheral blood from internal organs followed by 15 ml 4% (w/v) paraformaldehyde (pH 7.2). The lumbar spinal cord was carefully collected and fixed in 4% (w/v) paraformaldehyde overnight at 4℃. Paraffin-embedded sections were prepared. 5-µm-thick transverse paraffin sections were stained with Hematoxylin and Eosin (H&E) or Luxol fast blue (LFB, Sigma, S3382) for visualizing the number of infiltrating cells and myelin loss in inflammatory foci per section, respectively. Image-Pro software was utilized for quantifying levels of inflammation and demyelination, respectively.

### Isolation and analysis of CNS leukocyte infiltration

Brain and spinal cord isolated from 0.5% CMC and BrQ-treated EAE mice after perfusion with 0.9% NaCl solution on day 18 post-immunization were homogenized in ice-cold tissue grinders. Single-cell suspensions were obtained by filtering through 70-µm cell strainers and the cells were collected by centrifugation at 500×g for 10 min at 4 °C. The cells were resuspended in 8 ml 37% isotonic Percoll (GE Healthcare, USA) and overlaid onto 4 ml 70% isotonic Percoll cushion in 15 ml tubes. The gradient was centrifuged at 780×g for 20–30 min at 25 °C. Cells collected at 37/70% (vol/vol) Percoll interface were re-stimulated for 4–6 h at 37 °C in vitro and subjected to flow cytometry with cell surface, intracellular characteristic cytokine, or transcription factors staining. According to the manufacturer’s instructions, CountBright™ absolute counting beads (Invitrogen, C36950) were used to quantify the absolute number of total CNS leukocyte infiltration, as well as CNS infiltrating CD4^+^ T cells, including Th1, Th17 and Treg cells.

### CD4^+^ T cell isolation and in vitro Th1/Th17/Treg differentiation

Naive CD4^+^ T cells were prepared from the spleen of C57BL/6 mice (7–9 weeks of age) by magnetic depletion separation (BioLegend, 480040). Naive CD4^+^ T cells were cultured in complete RPMI 1640 containing 10% FBS, 2-mercaptoethanol (50 µM), L-glutamine (2 mM), 100 Units/mL penicillin and 100 µg/ml streptomycin. The cells were activated with anti-CD3 (2 µg/ml, BioLegend, 100331) and anti-CD28 (2 µg/ml, BioLegend, 102112). Anti-IL-4 (BioLegend, 504115, 10 µg/ml) and IL-12 (10 ng/ml) were added for Th1 polarization. For Th17 differentiation, the cells received anti-IL-4 (10 µg/ml) and anti-IFN-γ (BioLegend, 505810, 10 µg/ml) plus a Th17 mixture containing IL-6 (30 ng/ml), TGF-β1 (3 ng/ml), IL-23(10 ng/ml), TNF (10 ng/ml) and IL-1β (10  ng/ml). Polarization of Treg was induced by the addition of IL-2 (10 ng/ml), TGF-β1 (5 ng/ml) and anti-IFN-γ (10 µg/ml). BrQ at various concentrations was added to assess their influence on Th1, Th17, and Treg cell differentiation in vitro. Cells were collected for analysis after incubation for 3–4 days.

### Flow cytometry

CNS infiltrates, draining lymph node cells or CD4^+^ T cells from differentiation assays in vitro were stimulated with PMA (50 ng/ml, Sigma), ionomycin (750 ng/ml, Sigma), and brefeldin A (10 µg/ml, TargetMol) for 4–6 h at 37 °C. Surface markers (CD4, CD8, B220, and CD11b) were stained for 30 min in the dark at 4 °C with the appropriate antibodies, then the cells were washed and resuspended with 1xPBS. For intracellular cytokine IL-17 A and IFN-γ analysis, cells were fixed and permeabilized with an Intracellular Fixation & Permeabilization Buffer Set (eBioscience, 88-8824) according to the manufacturer’s protocol following surface staining. For Foxp3 staining, a Foxp3 staining buffer set (eBioscience) was used following the manufacturer’s instructions.

Cells were analyzed with CytoFLEX S System (Beckman Coulter). Flow cytometry data were plotted and quantified with FlowJo 10.8.1 software. The following antibodies were used: Pacific Blue™ anti-mouse CD4 antibody (Biolegend, 100531, 1:100), PE/Cyanine7 anti-mouse CD8a antibody (Biolegend, 100722, 1:100), APC anti-mouse/human CD45R/B220 antibody (Biolegend, 103212, 1:100), FITC anti-mouse/human CD11b antibody (Biolegend, 101206, 1:100), PE anti-mouse IL17a (Biolegend, 506904, 1:100), APC anti-mouse IFN-γ (Biolegend, 505810, 1:100), FITC anti-mouse Foxp3 (eBioscience, 11-5773-82, 1:100). Flow cytometry analysis was conducted by CytoFLEX flow cytometer (Beckman Coulter) and assessed with the FlowJo 10.8 software.

### Cytokine cytometric bead array (CBA) assay

The whole blood was collected from EAE mice treated with either 0.5% CMC solution or 30 mg/kg BrQ (administered orally, starting from day 3) on day 10 post-immunization. After collection, the blood samples were allowed to clot at room temperature for 30 min, followed by centrifugation at 1,000 g for 10 min at 4˚C to obtain the serum. Subsequently, the prepared supernatant was transferred to RNase/DNase-free tubes, aliquoted and stored at -80 °C until further analysis. The concentrations of IFN-γ, IL-17a, TNF, IL-10, and IL-6 in the serum were quantified using the Cytometric Bead Array Mouse Cytokine Kit (BD Biosciences, 560,485) according to manufacturer instructions. Cytometric bead array data were acquired by CytoFLEX flow cytometer (Beckman Coulter) using CytExpert software and analyzed using BD Cytometric Bead Array software (BD Biosciences).

### In vivo CD8^+^ T cells depletion

The EAE mice were randomly assigned to four groups in a blinded fashion: Isotype + Vehicle; Isotype + 30 mg/kg BrQ; anti-CD8 antibody + Vehicle; anti-CD8 antibody + 30 mg/kg BrQ (*n* = 6–8 per group). The anti-CD8 antibody (BioXcell, BE0004-1, Clone:53 − 6.7) was intraperitoneally administered to EAE mice at a dose of 100 µg on both day 3 and day 4 following immunization. Subsequently, a supplementary dose of 30 µg anti-CD8 antibody was administered once on day 11. Control animals received an equal dose of isotype antibody (BioXcell, BE0089, Clone:2A3). Careful attention was given to dosage and timing to ensure effective depletion of CD8^+^ T cells in EAE mice while maintaining mouse survival rates. The efficacy of the depletion was assessed through FACS analysis of peripheral blood T cells on days 10 and 15, respectively. The results revealed a clearance efficiency of 71.2% on day 10 and 58.3% on day 15 (Supplementary Fig. 3). Following CD8^+^ T cell depletion or BrQ treatment, the progression of EAE symptoms was observed to evaluate the contribution of CD8^+^ T cells in BrQ-mediated amelioration of EAE clinical disease severity.

### CFSE-based proliferation assays

Total leukocytes were obtained from the draining lymph nodes of 8-9-week-old C57BL/6 mice, primarily collected from the axillary, lateral axillary, and superficial inguinal regions. Naive CD8^+^ T cells were isolated using the MojoSort™ Mouse CD8^+^ T Cell Isolation Kit (Biolegend, 480035). The leukocytes and naive CD8^+^ T cells were then adjusted to a cell concentration of 1 × 10^6^ cells/mL with pre-warmed PBS solution. Next, an appropriate volume of CellTrace™ CFSE stock solution (Invitrogen, C34554, diluted at 1:2000) was added to the cell suspension to achieve a final working solution of 2.5 μM. Subsequently, the cells were incubated for 20 min at 37 °C in a water bath, protected from light. Following two washes, the cells were cultured in complete RPMI 1640 medium supplemented with 10% FBS, 2-mercaptoethanol (50 µM), L-glutamine (2 mM), 100 Units/mL penicillin, and 100 µg/ml streptomycin, anti-CD28 (5 µg/ml), anti-CD3 (5 µg/ml), and IL-2 (20 ng/ml). After 3 days of culture, CD8 surface staining was performed and CFSE-labeled cells were analyzed using flow cytometry to assess proliferation within the CD8^+^ gate.

### Bulk RNA-seq and data analysis

The lymphocytes in the dLN on day 10 after immunization were obtained from EAE mice treated with 0.5% CMC solution or 30 mg/kg BrQ (each group contains two pools, *n* = 3 mice per pool). After washing twice with 1xPBS, the cells were harvested for total RNA extraction by Trizol reagent (Invitrogen, 15596-018) following the manufacturer’s procedure. Then, the samples were qualified, 1–3 µg total RNA of each sample was used as the starting material to construct a transcriptome sequencing library according to the instructions of VAHTS Universal V6 RNA-Seq Library Prepkit for Illumina (NR 604-01/02). The cDNA sequencing on IlluminaHiseq4000 sequence platform was performed by Annoroad Gene Technology Co., Ltd (Beijing, China). The raw data were first under quality control processed with fastp (0.20.0). Subsequently, hisat2 (version 2.1.0) and htseq-count (version 0.11.2) were used to map the quality-controlled samples to the mouse reference genome and generate the gene count matrix. Differentially expressed genes were determined by DESeq2 package (Bioconductor) and only differentially expressed genes with fold change > 2 or fold change <-2 that were statistically significant (*p* value < 0.05) were selected.

### Cuprizone-induced demyelination mouse model

C57BL/6 mice (9 weeks) maintained in pathogen-free conditions were fed with 0.2% (w/w) cuprizone (Sigma) mixed with ground standard rodent chow. The cuprizone diet was maintained for 5 weeks, thereafter the cuprizone-infused feed was removed and the mice were given standard chow. The mice were orally administered BrQ (30 mg/kg/day) from the fifth week. The treatment lasted for 1, 2, or 3 weeks. The mice were subsequently anesthetized with 1.25% tribromoethanol and sacrificed. The brains were carefully removed, paraffin-embedded, sectioned, and stained the corpus callosum with LFB for histopathological analysis. Image-Pro plus software was used for quantization of the myelin loss in the corpus callosum per section.

### OPC differentiation and immunocytochemistry

Neural progenitor cell (NPC)-derived OPCs were induced by culture of cortical NPCs with OPC differentiation conditions. Briefly, the cerebral cortex was removed from the E14.5-E17.5 embryonic mouse brain and mechanically dissociated into single cells by filtering through 70-µm cell strainers placed on a 50 ml conical tube, and then the cells were collected by centrifugation at 500×g for 5 min at 4 °C. The cells were cultured in NPC medium (DMEM/F12 media, 1% B27, 1% N2, 20 ng/mL EGF, and 20 ng/mL FGF2 for 3–5 days until neurosphere formation. The neurospheres were dissociated into single cells with accutase until passage 3 (P3) NPCs and plated on poly-ornithine plus laminin-coated plates in OPC differentiation medium (DMEM/F12, 1% B27, 1% N2, 10 ng/ml bFGF, 10 ng/ml PDGF-AA) for 2 days. To induce OPC differentiation, the cells were cultured in the oligodendrocyte medium (DMEM/F12, 1% B27, 1% N2) with or without BrQ (0.31, 0.63, 1.25 or 2.5 µM). After differentiation for 4–6 days, the cells were fixed with 4% PFA for 15 min at room temperature. The cells were subsequently stained with anti-MBP antibody (Abcam, ab7349, 1:500) and the Alexa Fluor 647 secondary antibody (Invitrogen, A78947, 1:2000), Hoechst 33342 was used to identify cell nuclei. For cell imaging, we scanned four pictures per well and detected the nuclei and MBP-positive cells using a DMi8 microscope (Leica) and quantitative image analysis was performed by Image J software.

### Statistical analysis

A two-way ANOVA test was used to assess the significance of EAE clinical scores between the vehicle and BrQ-treated group throughout the disease course. Student’s t-test was applied for the other analyses. All data are expressed as the mean ± SEM. Only *p* values < 0.05 were considered statistically significant (*p < 0.05, **p < 0.01, ***p < 0.001, and **** *p* < 0.0001). Statistical analysis was performed using the software GraphPad Prism 9.0.

## Results

### BrQ ameliorates clinical symptoms of EAE mice

**Fig. 1:**
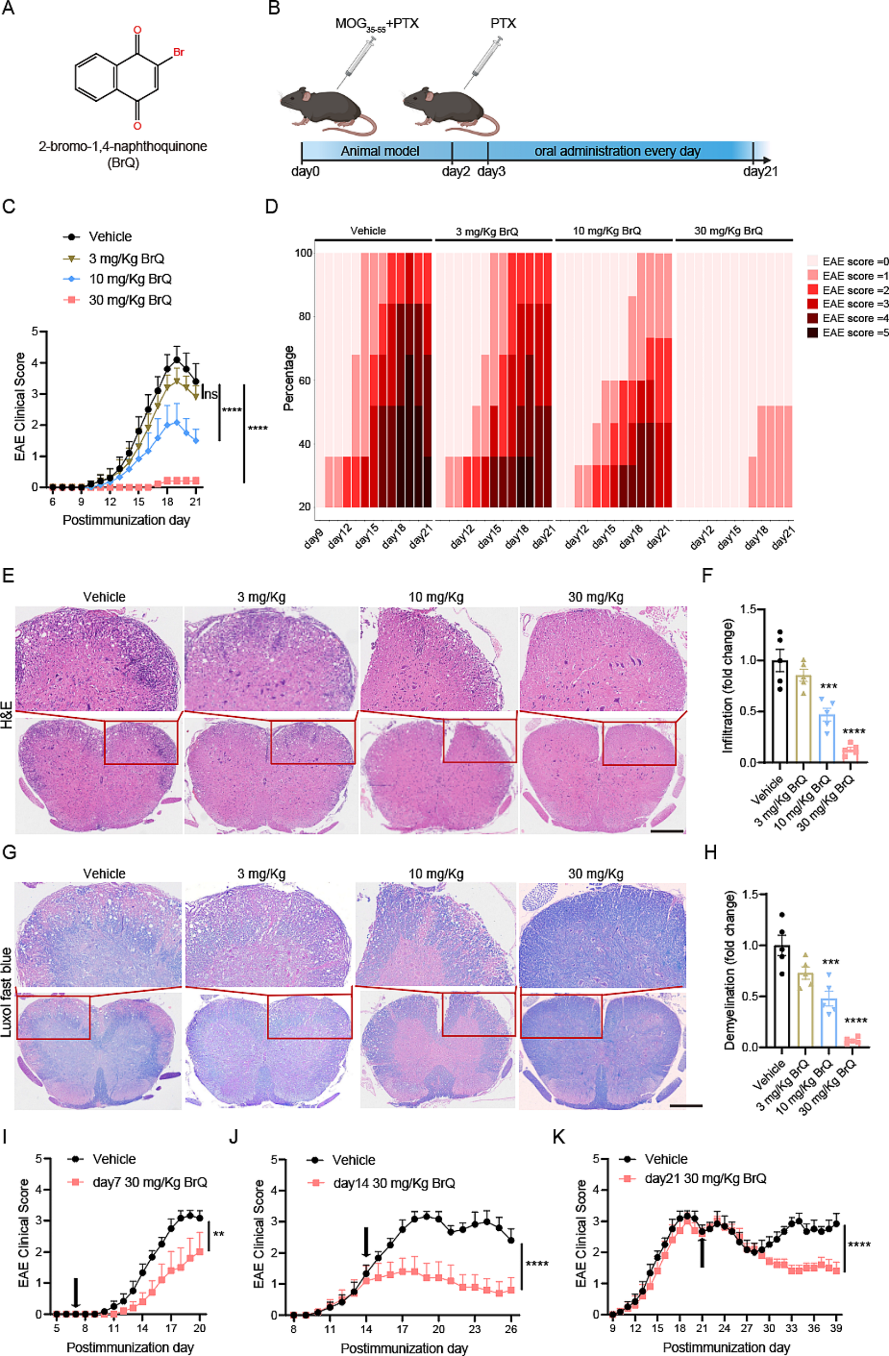
BrQ administration attenuates clinical symptoms of EAE mice. **A** Chemical structure of BrQ. **B** Schematic diagram of MOG_35 − 55_-induced EAE model and BrQ treatment. **C** Clinical scores of EAE mice treated with vehicle (0.5% CMC) or different dosages of BrQ (3, 10, or 30 mg/kg body weight) daily by gavage starting from day 3 after immunization, *n* = 5–6 mice per group. **D** The stacked column depicts EAE clinical incidence and severity from day 9 to day 21. **E**,** G** H&E staining and LFB staining of paraffin-embedded sections of spinal cords isolated from vehicle- or BrQ-treated EAE mice on day 18 post-immunization. Scale bars: 400 μm. **F**,** H** Statistical analysis of CNS inflammatory cell infiltration in H&E-stained sections and demyelination areas in luxol fast blue-stained spinal cord sections by Image-Pro software. *n* = 5 mice per group. **I-K** The disease scores of EAE mice treated with vehicle or BrQ (30 mg/kg) daily by gavage starting from day 7, day 14, or day 21 after immunization. *n* = 5–6 mice per group **p < 0.01, ****p < 0.0001 by two-way ANOVA. The experiments were repeated twice with consistent results

To investigate whether BrQ (Fig. 1A) confers protection against MS, MS animal model EAE was induced in male C57BL/6 mice by immunization with MOG_35–55_ peptide and 3,10 or 30 mg/kg BrQ was given every day via oral administration from day 3 after immunization, an equal volume (200 µL) of 0.5% CMC was given as vehicle control. The clinical score for EAE mice was assessed daily until the end of the experiment (Fig. 1B). The results showed that BrQ displayed a slight but statistically beneficial effect in alleviating disease severity at low dose of 3 mg/kg body weight. 10 mg/kg BrQ can markedly inhibit the disease severity of EAE and reduce disease incidence compared with the vehicle group. Encouragingly, 30 mg/kg BrQ administered by gavage resulted in a lower to nearly less than 50% morbidity, as well as a dramatically reduction in disease severity (Fig. 1C, D). Taken together, our results showed that BrQ not only markedly lowered the peak severity and cumulative clinical score of EAE mice in a dose-dependent manner but also delayed the onset and reduced disease incidence.

Histological analysis of spinal cord was performed at day 18 post-immunization. To assess leukocyte infiltration and myelin loss in the CNS, the lumbar spinal cords with the highest leukocyte infiltration and the most severe demyelination were acquired. We found that BrQ treatment leads to a dramatic reduction of inflammatory foci and leukocyte infiltration in the spinal cord than the vehicle group according to H&E staining result (Fig. 1E, F). Luxol fast blue staining also displayed less extensive demyelination in the white matter region of the spinal cord in BrQ-treated EAE mice (Fig. 1G, H).

To further gain insight into the mechanism underlying BrQ-mediated protection against EAE, we treated EAE mice with BrQ on different days during the disease progression until the end of this study. Our results indicated that treatment with BrQ, started before the onset of symptoms, significantly reduced the severity of the disease (Fig. 1I). More interestingly, an intervention protocol in which BrQ treatment was started after the onset of symptoms (day 14, when EAE clinical score reached 1), or the remission stage (day 21) also showed remarkable curative effect (Fig. 1J, K). Taken together, all these data demonstrate the preventative and therapeutic benefit of BrQ for EAE.

### BrQ does not promote OPC towards OL differentiation and myelin remyelination in cuprizone-induced demyelination mouse model

**Fig. 2:**
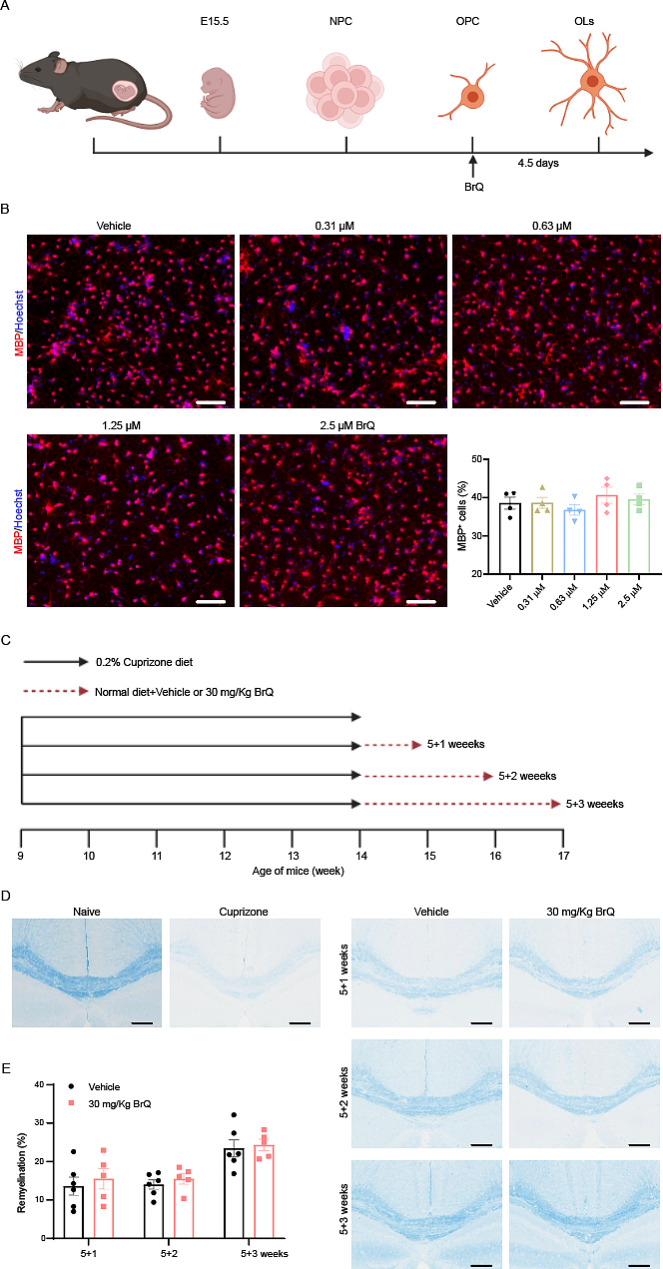
BrQ does not display any effect on OPC differentiation and myelin remyelination. **A** Schematic diagram for the generation of OLs from cortical NPCs isolated from mouse E15.5 embryos. **B** Representative immunofluorescent staining images of the OL marker MBP^+^ cells in OPCs induced to differentiate toward OLs in the presence of various concentrations of BrQ for 4.5 days. The percentage of MBP^+^ cells was quantified. Scale bars, 100 μm. **C** Schematic illustration of the cuprizone-induced demyelination mice model and BrQ treatment. Male adult C57BL/6 mice were induced demyelination by feeding with a diet that contained 0.2% (w/w) cuprizone for 5 weeks. Following cuprizone withdrawal, the mice were treated with 0.5% CMC solution or BrQ (30 mg/kg, oral administration) for 1, 2, or 3 weeks. **D** Representative images of the corpus callosum region stained with Luxol fast blue after cuprizone and BrQ treatment. Scale bars, 200 μm. **E** Statistical analysis of the myelinating areas in **D** (*n* = 5–6 mice per group). Data are shown as mean ± SEM and analyzed by unpaired two-tailed t-test. The experiments were repeated twice with consistent results

Considering that BrQ treatment had such a striking palliative effect on EAE mice, we wondered whether BrQ could simultaneously and directly promote myelin regeneration. Oligodendrocyte precursor cells (OPCs) migrate throughout the brain and spinal cord where they proliferate and differentiate into oligodendrocytes (OLs), OLs wrap nerve fibers with layers of specialized cell membrane to form myelin sheaths [20, 21]. Thus, we firstly established an assay to induce OL differentiation from neural progenitor cells (NPC)-derived OPCs to assess the effect of BrQ in promoting OPC differentiation towards OL (Fig. 2A). Mice E15.5 embryos were used to obtain cortical neurospheres, which could be propagated and passaged in NPC medium. NPCs were then induced into OPCs in OPC medium supplemented with bFGF and PDGF-AA. After removal of growth factors, OPCs differentiated spontaneously into OLs in the presence of different concentrations of BrQ for 4.5 days, and then the cells were fixed and subjected to immunofluorescence staining for MBP, a marker of mature OLs. The quantification data showed that the fraction of MBP^+^ cells did not increase with BrQ concentration, suggesting BrQ may have no effect on OL differentiation from OPC or myelin sheath repair in vitro (Fig. 2B).

To further evaluate the efficacy of BrQ in enhancing remyelination, we used the widely recognized cuprizone induced demyelination model [22]. Nine-week-old adult male C57BL/6 mice were fed 0.2% (w/w) cuprizone diet for 5 weeks, followed by standard chow and daily oral administration of BrQ (30 mg/kg) or vehicle for 1–3 weeks (Fig. 2C). The myelin status in the corpus callosum region was assessed by Luxol fast blue staining, a copper phthalide dyestuff, which has the property of binding to the myelin sheath in an alcoholic solution. The result showed that cuprizone treatment resulted in an almost complete loss of myelin in the corpus callosum compared with normal C57BL/6 mice. After cuprizone removal, spontaneous myelin repair could be gradually observed in the vehicle and BrQ groups at 1, 2 and 3 weeks. However, BrQ treatment did not substantially accelerated the remyelination course (Fig. 2D, E). Overall, The findings indicate that BrQ could not promote myelin regeneration both in vitro and in vivo.

### BrQ promotes activation and proliferation of CD8^+^ T cells in EAE mice

**Fig. 3:**
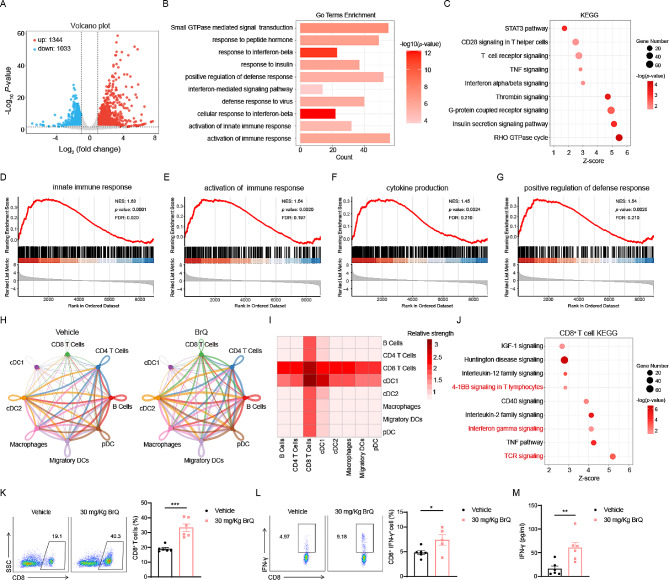
BrQ prominently activates and promotes the proliferation of CD8^+^ T cells in vivo. The leukocytes isolated from the dLN of EAE animals treated with 0.5% CMC solution or 30 mg/kg BrQ on day 10 post-immunization were performed bulk RNA-seq. **A** Volcano plot represents the differential expressed gene between BrQ and vehicle groups. Genes which were differed significantly compared to the vehicle group were colored with red (highly expressed in BrQ group) or blue (highly expressed in control group). **B** Gene ontology analysis of BrQ group significant gene. **C** KEGG analysis of BrQ group significant genes. **D-G** GSEA maps of the major differential gene sets in the vehicle and BrQ groups. **H**,** I** Circle plots and heatmap showing inferred cell-cell communications (ligand–receptor pairs) between any two immune cell subsets in dLN. Different colors in the circle plot represent different cell groups and the thickness of the line represents the strength of communication between the two cells, with thicker lines indicating stronger communication (interactions). In the heatmap, the vertical coordinate is the ligand, which corresponds to the arrow beginning cell type in the circle plots; the horizontal coordinate is the receptor, which corresponds to the arrow pointing to the cell type. The color in each cell represents the relative communication strength. **J** KEGG pathway analysis of CD8^+^ T cells from leukocytes isolated from the dLN. **K** Representative FACS images and statistics of CD8^+^ T cells in the leukocytes of dLN from EAE mice treated with BrQ (30 mg/kg) or 0.5% CMC solution on day 10 after immunization. **L** The expression of IFN-γ in the CD8 gate was analyzed by intracellular staining. Data are presented as mean ± SEM. *p < 0.05, ***p < 0.00 versus vehicle group (*n* = 5–6 per group, Student’s t test). The experiments were repeated 2–3 times with consistent results

To comprehensively explore the underlying mechanisms of BrQ-mediated amelioration of EAE symptoms, we performed bulk RNA-seq analysis of lymphocytes in the dLN on day 10 after immunization in the EAE mice treated with 0.5% CMC solution or 30 mg/kg BrQ. As shown in the volcano diagram (Fig. 3A), we identified 2347 differentially expressed genes compared to the vehicle group (fold change > 2, *p* < 0.05), among which 1344 genes were upregulated, and 1003 genes were downregulated. Further GO (Gene ontology) enrichment analysis revealed that the gene categories significantly affected by BrQ in the dLN lymphocytes cells were associated with activation of immune response, activation of innate immune response, response to interferon-beta, cellular response to interferon-beta and positive regulation of defense response (Fig. 3B). Similarly, T cell receptor signaling, CD28 signaling in T helper cells and Interferon alpha/beta signaling pathway also stood out according to KEGG (Kyoto Encyclopedia of Genes and Genomes) pathway analysis (Fig. 3 C). Consistent with the above GO and KEGG results, the GSEA (Gene Set Enrichment Analysis) analysis also showed that activation of immune response, cytokine production, innate immune response and positive regulation of defense response were upregulated, indicating that BrQ extensively activates immune cells in the dLN (Fig. 3D-G). We then employed CellChat analysis to investigate which immune cells are activated and contribute to alleviating EAE clinical symptoms. The results showed that activation of CD8^+^ T cells was most pronounced in peripheral dLN lymphocytes, primarily collected from the axillary, lateral axillary, and superficial inguinal regions, in BrQ-treated EAE mice. Of note, we discovered that among these immune cells interacting with CD4^+^ T cells, CD8^+^ T cells were the most significantly altered signaling sender and receiver (Fig. 3H, I). In addition, QIAGEN Ingenuity Pathway Analysis (IPA) confirmed that CD8^+^ T cells activation associated signaling pathways, including TCR signaling and interferon gamma signaling, were elevated (Fig. 3J). Consistent with the above findings, our results demonstrated a substantial increase in the percentage of CD8^+^ T cells and CD8^+ ^IFN-γ^+^ T cells in the dLN of EAE mice in vivo, compared to the vehicle group (Fig. 3K, L). Moreover, BrQ administration significantly increased the levels of IFN-γ in serum (Fig. 3 M), while markedly reducing IL-17a levels, without affecting the levels of IL-6, TNF, and IL-10 (Supplementary Fig. 2). Altogether, these data demonstrate that BrQ promotes the remarkable activation and proliferation of CD8^+^ T cells in EAE mice.

### The amelioration of EAE clinical symptoms by BrQ relies on CD8^+^ T cells

**Fig. 4:**
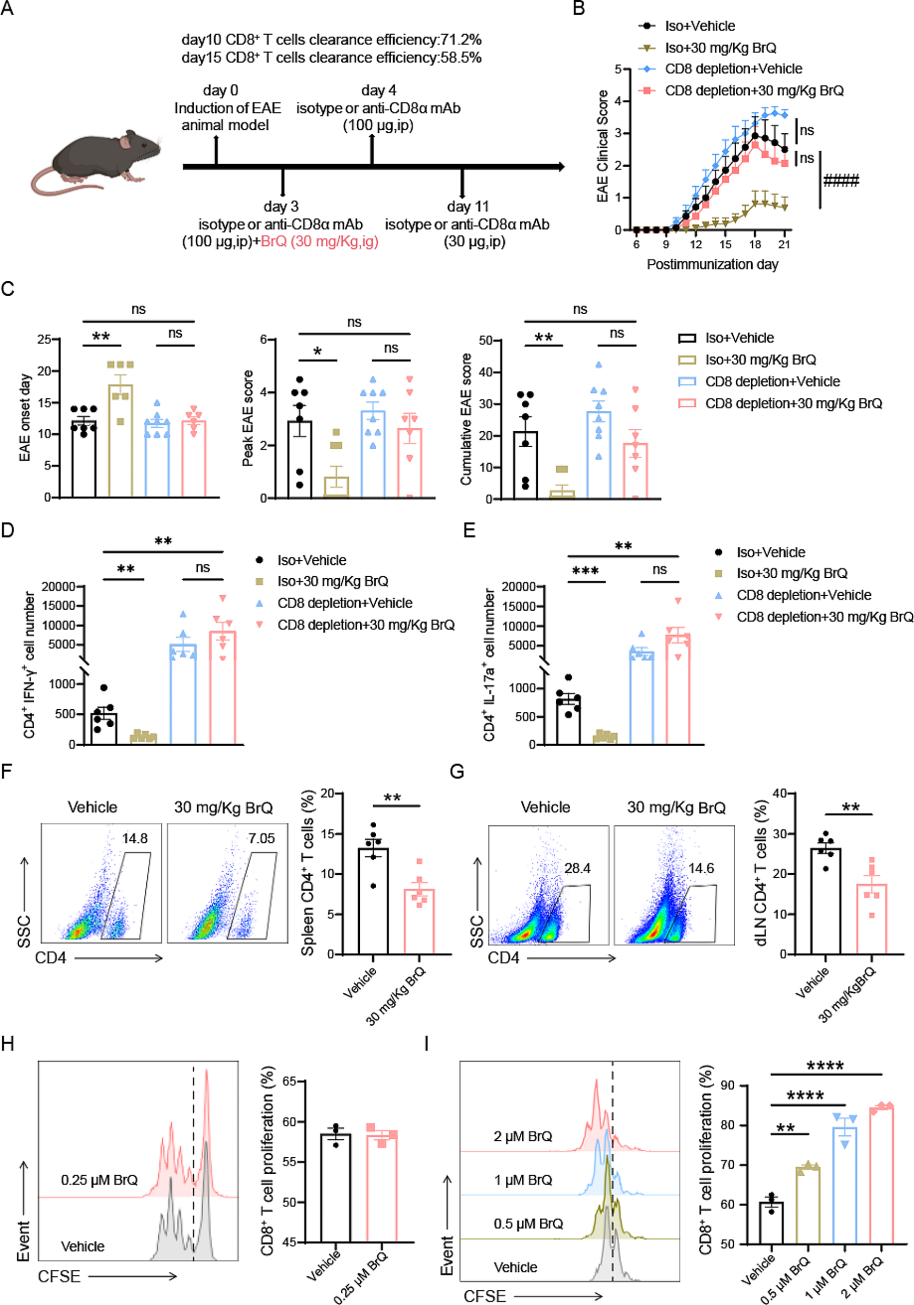
BrQ alleviates EAE symptoms in a CD8^+^ T cell-dependent manner. **A** Diagram illustrating the administration scheme of anti-CD8 or an isotype control in EAE mice. The EAE mice were treated daily by gavage with either 200 µL of vehicle (0.5% CMC) or 30 mg/kg of BrQ, starting from day 3 post-immunization. **B** The EAE clinical scores were assessed in mice treated with vehicle or BrQ (30 mg/kg), with or without CD8 depletion. ^####^*p* < 0.0001 by two-way ANOVA. **C** Statistical analysis was performed on the EAE onset day, peak EAE score, and cumulative clinical scores (*n* = 6–8 per group). Data are mean ± SEM. *p < 0.05, **p < 0.01 (one-way ANOVA test). **D-E** The number of Th1 and Th17 cells infiltrating the CNS were quantified in mice treated with vehicle or BrQ (30 mg/kg), with or without CD8 depletion, *n* = 6 per group. Data are mean ± SEM. **p < 0.01, ***p < 0.001, (one-way ANOVA test). **F-G** The splenocytes and leukocytes harvested from EAE mice on day 10 postimmunization were restimulated with MOG_35–55_ (20 µg/ml) for 48 h and 72 h in vitro, respectively. Evaluation of recall responses of antigen-specific CD4^+^ T cell was performed using FACS analysis (*n* = 6). Data are mean ± SEM. **p < 0.01 (Student’s t test). **H** Isolated CD8^+^ T cells from the draining lymph nodes of C57BL/6 mice were labeled with CFSE. The proliferation of CD8^+^ T cells was assessed via flow cytometry after treatment with 20 ng/ml IL-2, 5 µg/ml CD3/CD28, and 0.25 µM BrQ for 3 days. **I** The total leukocytes from C57BL/6 mice were labeled with CFSE. The proliferation of CD8^+^ T cells was evaluated by flow cytometry after treatment with 20 ng/ml IL-2, 5 µg/ml CD3/CD28 and varying concentrations of BrQ for 3 days. Data are mean ± SEM. **p < 0.01, ****p < 0.0001 versus vehicle group by one-way ANOVA test. The experiments were repeated twice with consistent results

To determine how BrQ affected the inflammatory process in EAE, we analyzed the percentages of CD4^+^ T cells, CD8^+^ T cells, B cells, or macrophages in the spleen and dLN at day 9 postimmunization by flow cytometry. The results unveiled that the most prominent variations occurred in the percentages of CD8^+^ T cell subpopulations (Supplementary Fig. 1). Next, we aimed to explore the role of CD8^+^ T cells in BrQ-mediated alleviation of EAE clinical symptoms. We used a CD8-specific monoclonal antibody (CD8 mAb) to deplete CD8^+^ T cells after immunization. Injection on EAE day 3 and day 4 rapidly depleted CD8^+^ T cells, maintaining a 71.2% clearance rate even after 6 days (almost complete clearance of CD8^+^ T cells would lead to nearly complete mortality in mice during the progression of EAE, particularly at the peak of the disease). An additional dose was given on EAE day 11. The clearance efficiency of CD8^+^ T cell in peripheral blood on EAE day 15 was 58.5% with minimal impact on CD4^+^ T cells, as determined by flow cytometry (Fig. 4A and Supplementary Fig. 3). Our results revealed that CD8^+^ T cell depletion led to a slight exacerbation of EAE clinical symptoms. Importantly, the beneficial effects of BrQ disappeared after CD8 depletion, indicating that BrQ alleviates EAE in a CD8^+^ T cell-dependent manner (Fig. 4B, C). Notably, BrQ also reduces Th1 and Th17 cell infiltration into the CNS in a CD8^+^ T cell-dependent manner (Fig. 4D, E). To test whether the active suppression induced by expanded CD8^+^ T cells was directed toward antigen-specific CD4^+^ T cells. We isolated lymphocytes from the dLN and spleen of EAE mice treated with 0.5% CMC solution or 30 mg/kg BrQ on day 10 after immunization and examined the recall responses of these cells to MOG_35 − 55_ in vitro. Our results confirmed that the percentages of MOG_35 − 55_-reactive CD4^+^ T cell subpopulation in the dLN and spleen were dramatically reduced compared with the vehicle group (Fig. 4F, G).

To further investigate whether BrQ directly promotes CD8^+^ T cell proliferation, we obtained lymphocytes from dLN, isolated CD8^+^ T cells by negative selection, and labeled with CFSE. Then, different concentrations of BrQ were added and CD8^+^ T cell proliferation was assessed after culture for 3 days (cell apoptosis began to occur at 0.5 µM BrQ). The results showed that BrQ did not directly promote CD8^+^ T cell proliferation (Fig. 4H). However, the addition of BrQ to total lymphocytes significantly promoted CD8 expansion, suggesting that BrQ might induce CD8^+^ T cell proliferation indirectly by acting on other immune cells (Fig. 4I). In summary, our findings demonstrate that BrQ promotes CD8^+^ T cell proliferation indirectly, leading to the suppression of antigen-specific CD4^+^ T cells and ultimately alleviating EAE in a CD8^+^ T cell-dependent manner.

### BrQ restricts Th1 and Th17 development in vivo

**Fig. 5:**
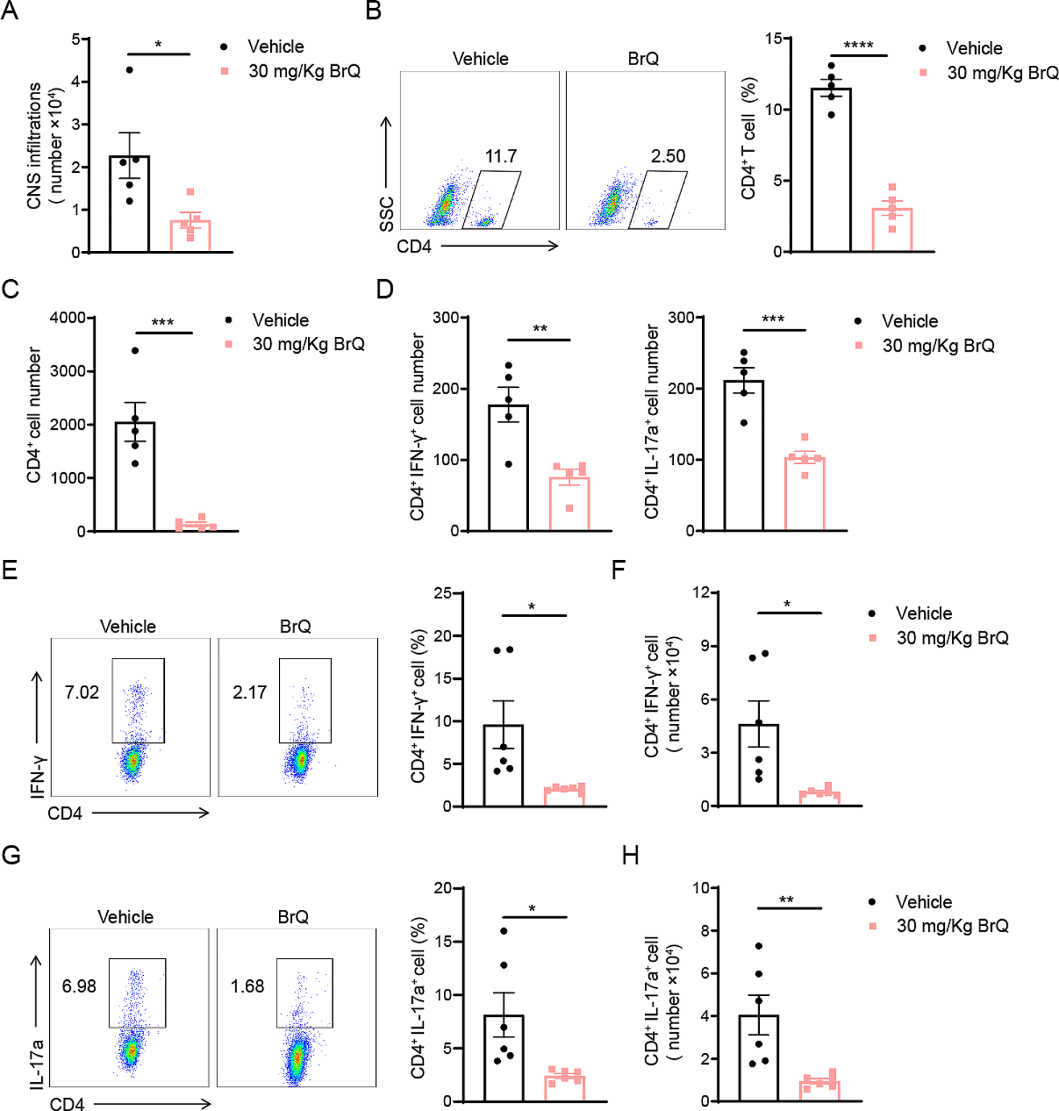
BrQ blocks Th1 and Th17 cells in EAE mice. **A** Total CNS infiltrate lymphocytes were isolated from EAE mice treated with 0.5% CMC solution or BrQ (30 mg/kg, oral, starting from day 3) on day 18 post-immunization and quantified by flow cytometry. **B** Flow cytometric analysis of CD4^+^ T cells in the CNS infiltrate lymphocytes. **C** Statistical data of absolute numbers of infiltrating CD4^+^ T cells within the CNS. **D** Absolute numbers of Th1 and Th17 cells in the infiltrated CD4^+^ T gate. **E**,** G** Leukocytes were isolated from the draining lymph node (dLN) of EAE animals treated with 0.5% CMC solution or 30 mg/kg BrQ on day 10 post-immunization. Th1 and Th17 cells were analyzed by intracellular staining of IFN-γ and IL-17 A in the CD4 gate, respectively. **F**,** H** Statistical analysis of absolute numbers of Th1 and Th17 cells in dLN (*n* = 5–6 per group). Data represent mean ± SEM. *p < 0.05, **p < 0.01, ***p < 0.001, ****p < 0.0001versus vehicle group (Student’s t test). The data are a representative for two independent experiments

Moreover, Th1 and Th17 cells are long considered to be the major effectors and culprits in a plethora of autoimmune and inflammatory diseases including multiple sclerosis [23–25]. Thus, we turned our attention to explore whether BrQ alleviated the disease severity of EAE mice through activated CD8^+^ T cells mediated Th1 and Th17 inhibition in vivo. Firstly, we analyzed the number of inflammatory cells infiltrating into the CNS at day 18 post-immunization. The results demonstrated that BrQ led to a significant decrease in total leukocyte infiltration in the CNS compared with the vehicle group (Fig. 5A). We further assessed infiltrating CD4^+^ cell in the spinal cord and brain by FACS analysis. Consistent with the above observation on total leukocyte infiltration in EAE mice, BrQ treatment also caused a remarkable reduction in CD4^+^ T cell number and percentage (Fig. 5B, C). In addition, the number of CD4^+^IFN-γ^+^ and CD4^+^IL17A^+^ T cells in the BrQ treated group were less than in vehicle treated mice (Fig. 5D). However, although BrQ promoted the proportion of Treg cells infiltrating the CNS, the absolute number of Treg cells did not increase due to the reduced total number of CNS-infiltrating cells (Supplementary Fig. 5C, D). These findings indicated that BrQ treatment reduced the infiltration of inflammatory cells into the central nervous system, especially the number of Th1 and Th17 cells.

Furthermore, we analyzed the profile of MOG-reactive CD4^+^ T cells in the peripheral draining lymph node of BrQ-treated and untreated mice at day 10 post-immunization. The results revealed that the proportion of Th1 and Th17 cells in the CD4^+^ population were dramatically reduced (Fig. 5E, G). Consistent with the observation in the CNS, BrQ treatment also caused a remarkable decrease in the total number of Th1 and Th17 cells in the lymphocytes of the draining lymph node (Fig. 5F, H). Moreover, BrQ significantly increased the proportion and total number of Treg cells in the dLN (Supplementary Fig. 5A, B). Nevertheless, FACS analysis revealed that BrQ did not influence the differentiation of Th1, Th17, or Treg in vitro (Supplementary Fig. 4). Taken together, these data drive us to conclude that BrQ reduces Th1 and Th17 frequencies and EAE immunopathology by promoting CD8^+^ T cell activation and proliferation.

### No immunotoxicity signs were observed in BrQ-treated C57BL/6 mice

To assess the safety of BrQ administration in vivo, we assessed the safety of BrQ in vivo. Normal male C57BL/6 mice (7–8 wk) were gavaged with 200 µL 0.5% CMC solution or different dosages of BrQ (3, 10 or 30 mg/kg) daily for 3 weeks and their body weight was weighed weekly.

**Fig. 6:**
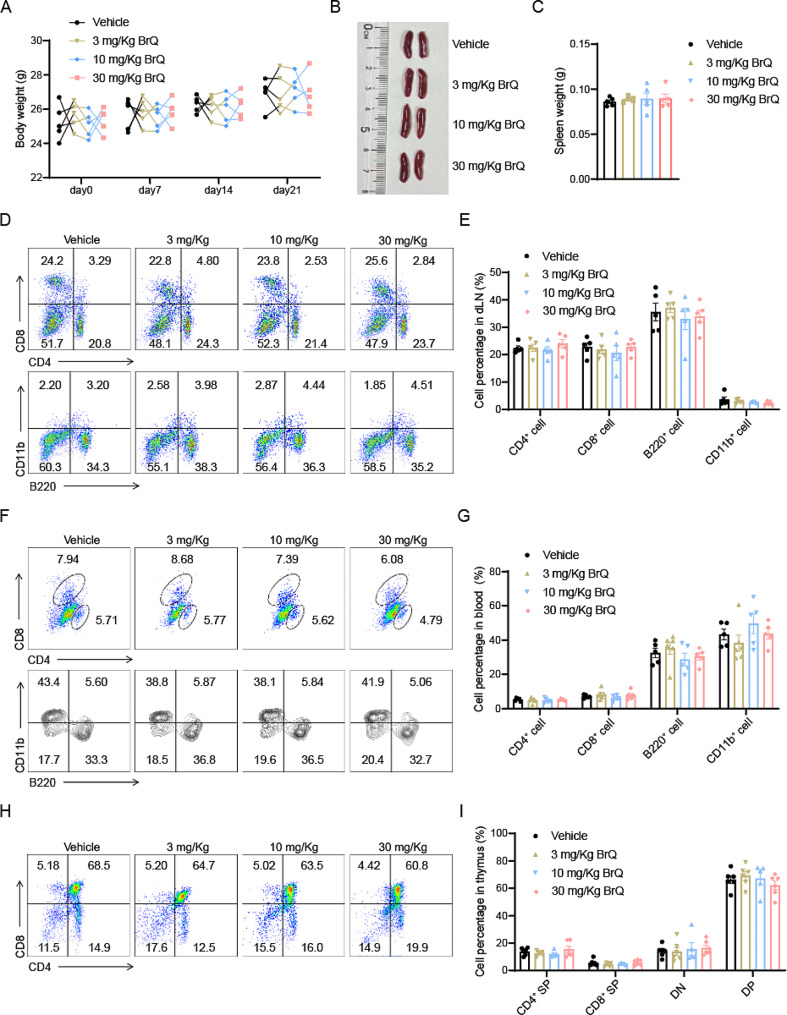
No signs of immunotoxicity are observed in BrQ-treated mice. Normal 8-week-old C57BL/6 mice were administered orally with 200µL vehicle (0.5% CMC) or BrQ (3, 10, or 30 mg/kg/day) for 21 days. **A** Body weight was monitored every week. **B**,** C** Spleen samples were collected, pictured and weighed. **D**,** E** Leukocytes in the draining lymph node were isolated and stained for CD4^+^ T cells, CD8 + T cells, B cells (B220) and CD11b^+^ cells. Cell percentages were determined with flow cytometry analysis. **F**,** G** Cell surface staining results and statistic data of the CD4^+^ T cells, CD8^+^ T cells, B cells (B220) and CD11b^+^ cells in the blood. **H**,** I** Flow cytometric analysis and quantification of CD4^+^, CD8^+^, CD4^−^CD8^−^ (double negative, DN) and CD4^+^CD8^+^ (double positive, DP) in the thymus. Data are mean ± SEM of 5 mice per group versus vehicle group (one-way ANOVA). Representative data of two independent experiments

The data showed there were no significant alterations in C57BL/6 mice treated with various dosages of BrQ (Fig. 6A). Moreover, there were no discernible changes in spleen size, morphology, or weight (Fig. 6B, C), indicating that BrQ administration at the macroscopic level had no significant effect on normal growth and spleen health in mice.

Considering that the lymphatic and circulatory systems are critical in maintaining immunological stability and safeguarding the body from disease [26]. We next validated whether the administration of BrQ had any toxic effects on the major immune cell populations in the draining lymph nodes and bloodstream. Leukocytes from the draining lymph node and blood of mice were collected after the BrQ administration continued for 21 days and subjected to flow analysis for CD4, CD8, B220, and CD11b cells to differentiate among T cells, B cells, and macrophages/neutrophils. The results showed that the percentage of these cells remained unaltered (Fig. 6D-G). It has been recognized that T cells, particularly CD4^+^ T cells, play a crucial role in MS [27]. Furthermore, given that the thymus provides a nurturing site for the differentiation, development, and selection of T cells [28, 29], the effect of BrQ on T cell development in the thymus was also evaluated. As a result, the percentage of CD4^+^, CD8^+^, CD^+^CD8^+^ (DP) and CD4^−^CD8^−^ (DN) in the thymus also remained unchanged (Fig. 6H, I). These observations lead us to conclude that BrQ did not exert significant immunotoxicity and provided strong support for its safety in further research and potential clinical applications. Altogether, these data suggested that BrQ administration induces negligible immunotoxicity, supporting its safety in further research and potential clinical applications.

## Discussion

**Fig. 7:**
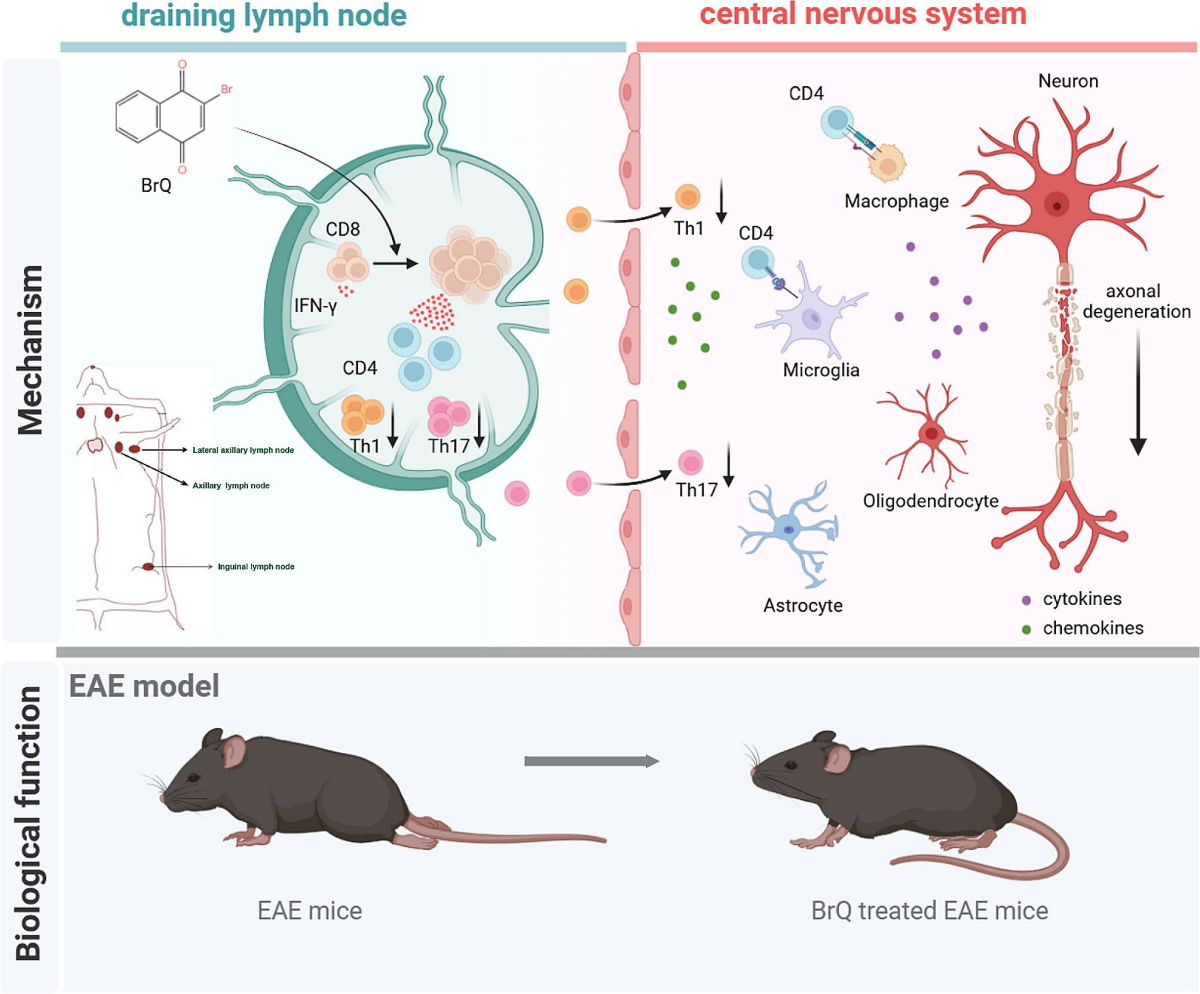
Schematic diagram of the protective mechanism of BrQ against EAE pathogenesis

As the most prevalent non-traumatic neurological disease among young adults, MS has been a rapidly growing global health concern [30]. Despite the introduction of various disease-modifying therapies in recent years, a complete clinical cure for MS remains elusive, with currently available treatments only capable of slowing disease progression. Consequently, it is urgently needed to develop safe and efficacious drugs targeting multiple sclerosis [31]. In our study, we observed that mice treated with 2-bromo-1,4-naphthalenedione (BrQ), a derivative of naphthalenedione, exhibited reduced severity of EAE symptoms, including lower clinical scores, decreased leukocyte infiltration, and less extensive demyelination in the CNS. Through comprehensive analysis of cell interactions using bulk RNA-seq data and validation through flow analysis, we discovered that BrQ significantly enhances the expansion of CD8^+^ T cells and their interactions with other immune cells in peripheral immune tissue in EAE mice. Further experiments involving CD8^+^ T cell depletion confirmed that BrQ alleviates EAE in a CD8^+^ T cell-dependent manner. Mechanistically, we found that expanded CD8^+^ T cells selectively reduce antigen-specific CD4^+^ cells, subsequently inhibiting the development of Th1 and Th17 cells in vivo, ultimately leading to relief from EAE. Our findings shed light on the unique therapeutic potential of BrQ for the treatment of MS (Fig. 7).

Substantial evidence suggests that CD8^+^ T cells are the primary immune cell population within CNS lesions of MS patients, indicating their pivotal role in the disease progression of MS [27, 32]. Both in MS patients and the EAE animal model, clonally expanded CD8^+^ T cell populations effectively reduce disease severity by actively suppressing autoreactive CD4^+^ T cells. Conversely, depletion of CD8^+^ T cells exacerbates EAE symptoms [33–35]. Moreover, nonclassical MHC class Ib–restricted CD8^+^ T cell (HLA-E in humans and Qa-1b in mice), CNS-specific CD8^+^ T, CD8^+^CD25^+^Foxp3^+^, CD8^+^CD44^+^Ly49^+^, CD8^+^CD28^−^, CD8^+^CD122^+^ and KIR^+^CD8^+^ T cells have been suggested to have a regulatory function [36–39]. In contrast, IL-17 A secreting CD8^+^ T cells and myelin antigen-specific CD8^+^ T cells were demonstrated to support EAE symptoms [40, 41]. Given the heterogeneity and complexity of CD8^+^ T cell subpopulations, none of the twenty-three disease-modifying therapies approved by the U.S. Food and Drug Administration was specifically developed to target CD8^+^ T cells for treating MS. Although glatiramer acetate mitigates EAE to some extent by inducing Qa-1-restricted regulatory CD8^+^ T cells. Notably, CD8^+^ T cells were identified as primary signaling mediators in the peripheral immune system of BrQ treated EAE mice. Importantly, we observed that BrQ activates and amplifies CD8^+^ T cells within the spleen, dLN and blood, leading to a significant reduction in the MOG_35 − 55_-reactive CD4^+^ T cell subset in EAE mice. CD8^+^ T cell depletion confirmed the efficacy of BrQ was exerted in a CD8^+^ T cell-dependent manner. Furthermore, we observed the activation of 4-1BB signaling in CD8^+^ T cell after BrQ treatment, while the agonistic anti-4-1BB monoclonal antibody can suppress antigen-specific CD4^+^ T cells by activating CD8^+^ T cells in rheumatoid arthritis animal models [42]. We inferred that BrQ largely promotes the proliferation of CD8^+^ T cells by activating 4-1BB. Likewise, despite reduced CD8^+^ T cell levels in the blood on day 15 in EAE mice, asymptomatic BrQ-treated mice remained symptom-free even after BrQ administration cessation for 1 month (unpublished observation). We speculated that expanded CD8^+^ T cell not only exerts immunosuppressive effects but also potentially develop into antigen-specific memory T cell. Thus, BrQ may represent the first small molecule candidate drug capable of specific increasing CD8^+^ T cells with suppressive function, thereby providing novel therapeutic strategies for EAE and other autoimmune diseases.

It has been well established that activation of peripheral autoreactive effector CD4^+^ T cells mediate disease progression and severity [42]. Moreover, both IL-17 A-producing Th17 cells and IFN-γ-producing Th1 cells are recognized as pivotal players in driving the pathogenesis of EAE [43]. Upon activation, CD8^+^ T cells produce substantial amounts of IFN-γ, allowing them to modulate autoimmune responses by suppressing Th1 and Th17 cells in an IFN-γ-dependent manner [44]. Our results showed that BrQ boosts IFN-γ production in CD8^+^ T cells in vivo, along with increased serum levels of IFN-γ. As reported, IFN-γ deficiency exacerbates EAE [45]. The function of IFN-γ in regulating Th1 and Th17 responses have been well elucidated. First, IFN-γ limits Th1 responses by converting IFN-γ-producing Th1 cells into IL-10-producing type 1 regulatory T (Tr1) cells [46]. Secondly, IFN-γ directly inhibits naive T cells from differentiating into Th17 cells. Instead, the inhibition of IFN-γ signaling enhances pathogenic Th17 effector cell development, worsening autoimmunity [47]. IFN-γ also indirectly inhibits the pathogenic Th17 response by inducing dendritic cells to secrete IL-27 [48]. Thirdly, coculture with activated CD8^+^ T cells markedly enhances apoptosis of Th1 and Th17 cells in vitro. Additionally, activated CD8^+^ T cells can directly eliminate effector/autoreactive CD4^+^ cells by releasing cytolytic granules like granzyme B and perforin, leading to direct lysis [37, 44]. Furthermore, our research supports these findings by demonstrating that BrQ restrains Th1/Th17 cell development in both peripheral dLN and CNS in vivo. Collectively, our study suggests BrQ mediates the activation and expansion of CD8^+^ T cells to suppress Th1/Th17 development, thereby conferring protection against EAE/MS.

MS are characterized by autoimmune-mediated demyelination and neurodegeneration. The treatment of multiple sclerosis currently has two main goals: controlling the inflammatory activity and promoting remyelination. Of these, remyelination is a promising strategy for treating MS as it restores damaged CNS regions [49–51]. Accordingly, we next verified whether BrQ could directly promote myelin regeneration in vivo and in vitro. Our study indicated that BrQ does not stimulate OPC differentiation into mature oligodendrocytes that remyelinate axons and myelin sheaths for remyelination in vitro, nor does it have a direct effect on remyelination in a cuprizone-induced demyelination mouse model. We speculated that BrQ appears to modulate EAE pathology through an immunosuppressive effect rather than by directly promoting myelin regeneration. However, the inflammatory state of the peripheral immune system contributes to inefficient remyelination in MS [52]. Hence, BrQ, with its unique mechanism and notable efficacy, offers a promising approach when combined with agents promoting remyelination to achieve the goal of curing MS.

However, our study has several limitations. Firstly, BrQ does not directly promote CD8^+^ T cell proliferation, but its effects on immune metabolic stability, cytokine production, and functionality need further investigation. Secondly, it’s unclear whether BrQ-activated CD8^+^ T cells require IFN-γ to inhibit Th1 and Th17 frequencies and the exact mechanisms by which CD8^+^ T cells limit the encephalitogenic response in BrQ-treated EAE. Thirdly, although our findings indicate that BrQ promotes the proliferation of CD8^+^ T cells indirectly in vitro. However, it remains uncertain whether BrQ is metabolized into other substances in vivo or undergoes metabolic transformation by intestinal microorganisms into different products, directly promoting the proliferation of CD8^+^ T cells or alternatively exerting a protective effect on EAE by modulating intestinal immune cells. Lastly, the timing of BrQ’s effectiveness on EAE varies at different disease stages. Aside from BrQ’s potentially unique mechanism of action, we speculate that differences in underlying pathology, particularly the differential response rates of CD8^+^ T cells at various stages of the disease, result in varying responsiveness to BrQ at different stages. This will be the focus of our forthcoming research.

In summary, our study demonstrates that BrQ could be a unique molecule drug to amplify specially CD8^+^ T cells with suppressive function. These expanded CD8^+^ T cells specifically suppress pathogenic CD4^+^ T cells and Th1/Th17 development, ultimately resulting in relief from EAE. Thus, BrQ might present a promising treatment for MS and other autoimmune diseases in the future.

### Electronic supplementary material

Below is the link to the electronic supplementary material.

Supplementary Material 1

## Data Availability

No datasets were generated or analysed during the current study.

## Abbreviations

BBB: Blood-brain barrier
BrQ: 2-bromo-1,4-naphthalenedione
CNS: Central nervous system
dLN: draining lymph nodes
EAE: Experimental autoimmune encephalomyelitis
H&E: Hematoxylin and eosin
LFB: Luxol Fast Blue
MOG: Myelin oligodendrocyte glycoprotein
MS: Multiple sclerosis
OPC: Oligodendrocyte precursor cells
TCMs: Traditional chinese medicines
Th1: T helper 1 cells
Th17: T helper 17 cells
Treg: regulatory T cells
