# Supplementary material for: 2-Bromo-1,4-Naphthalenedione promotes CD8+ T cell expansion and limits Th1/Th17 to mitigate experimental autoimmune encephalomyelitis
Source: J Neuroinflammation. 2024 Jul 27;21:181. doi: 10.1186/s12974-024-03172-x (PMC11283727; doi:10.1186/s12974-024-03172-x)
Supplement: Supplementary file 1 — Supplementary Material 1 [file 12974_2024_3172_MOESM1_ESM.docx]

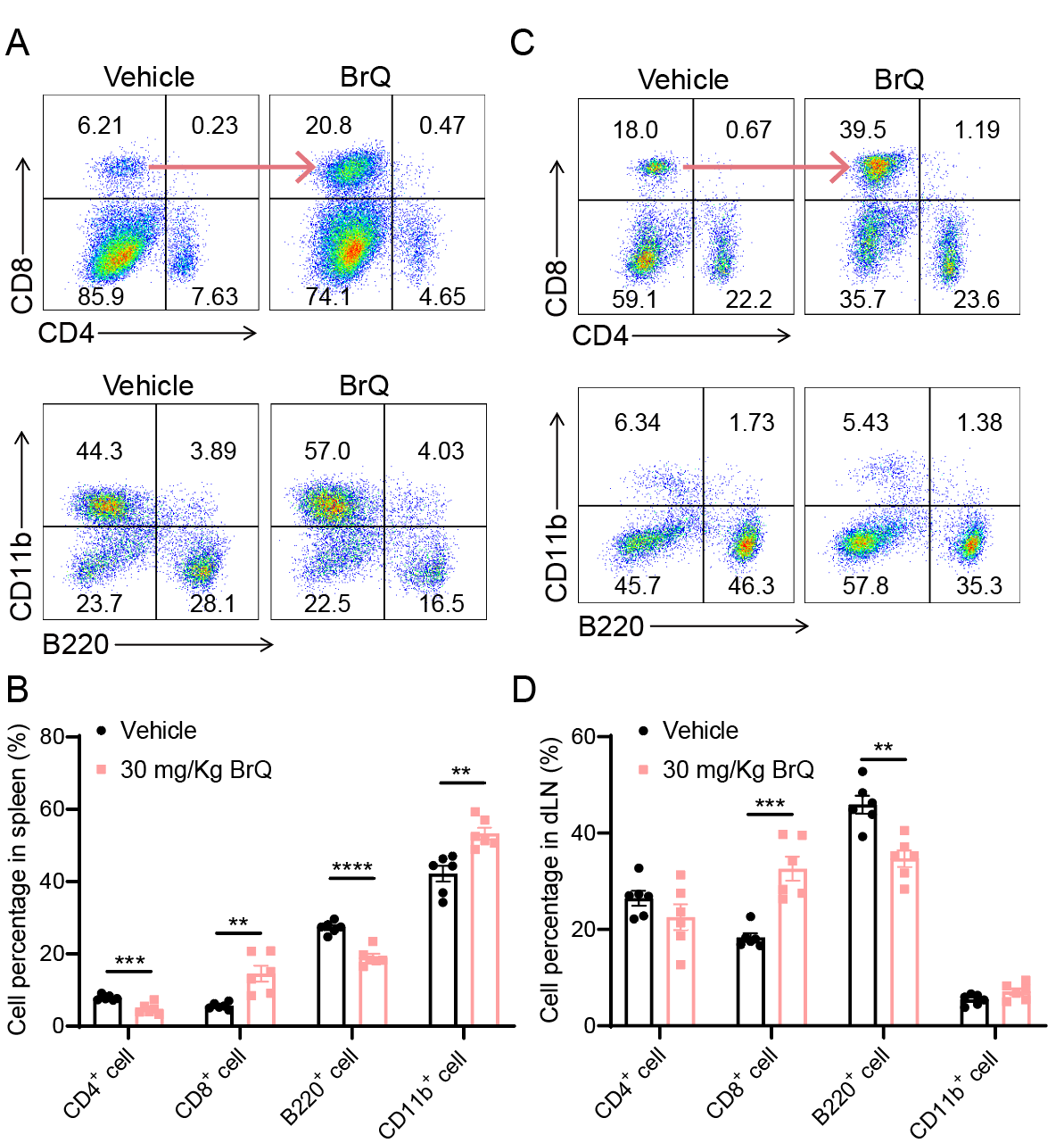


**Fig S1. The impact of BrQ on various subsets of mononuclear cells in EAE mice.** The total numbers of mononuclear cells in the spleen and draining lymph nodes were isolated from EAE mice treated with 30 mg/kg BrQ or vehicle on day 10 post immunization, n=6 per group. **A-B** The representative flow cytometry plots of CD4, CD8, B220 and CD11b expression in the splenocytes and cell percentages were determined. **C-D** The representative flow cytometry plots of CD4, CD8, B220 and CD11b expression in the leukocytes and cell percentages were determined. Statistical significance was determined as ***p* < 0.01, ****p* < 0.001, *****p* < 0.0001 by two-tailed unpaired Student’s t test. Representative data of two independent experiments.


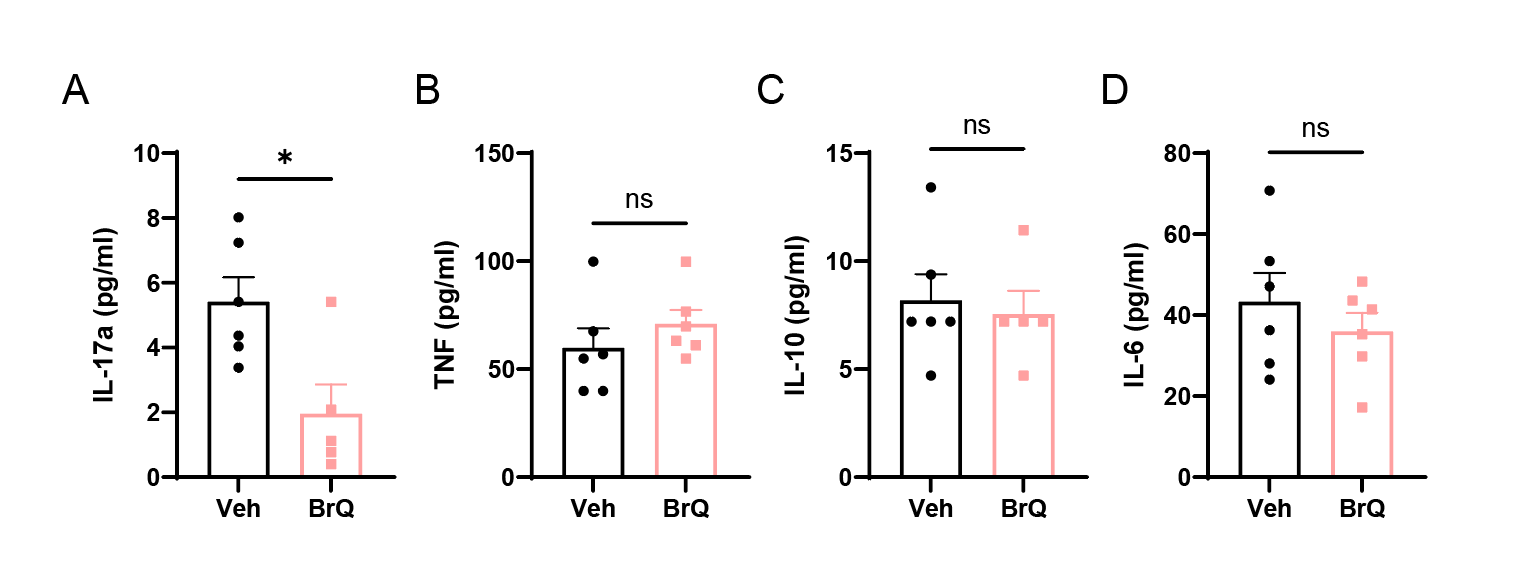


**Fig S2. The serum cytokine levels in EAE mice on day10 after immunization**. The serum was collected from EAE mice treated with 0.5% CMC solution or BrQ (30 mg/kg, oral, starting from day3) on day 10 post-immunization, n=5-6 per group. The levels of **(A)** IL-17a, **(B)** TNF, **(C)** IL-10**,** and **(D)** IL-6 were determined by cytometric bead array. Statistical significance was determined as **p* <0.05 by two-tailed Student’s t test.


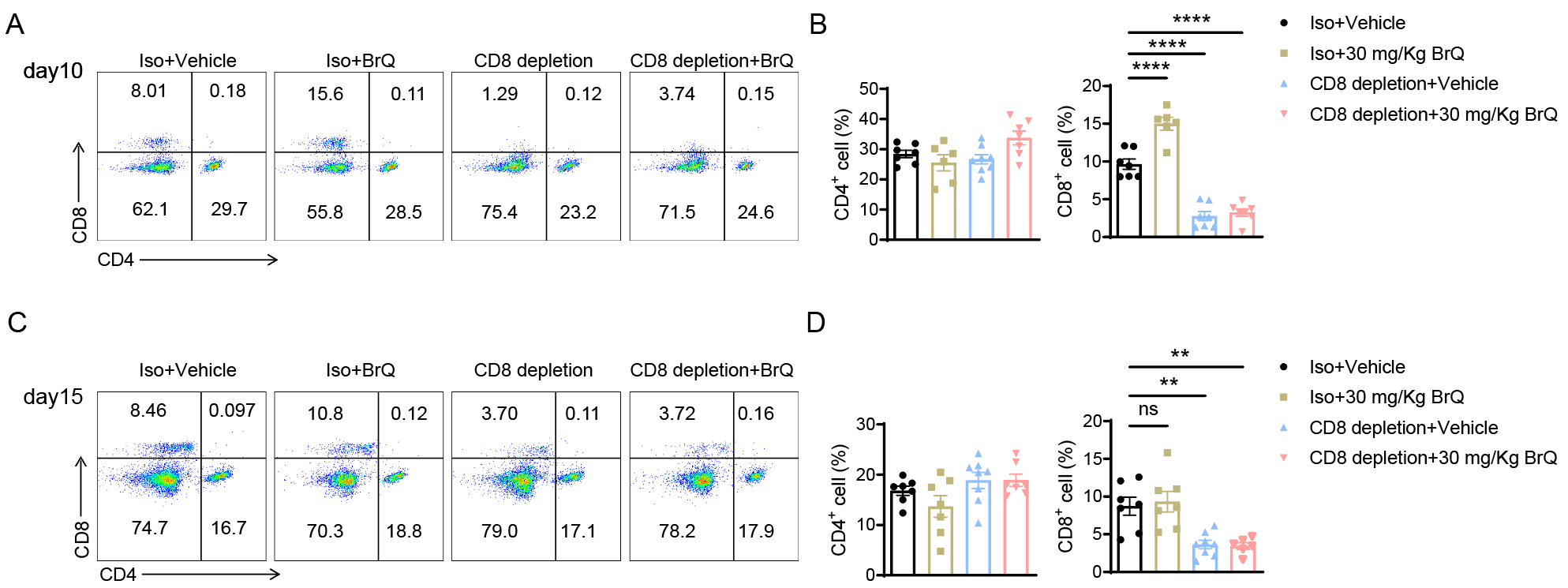


**Fig S3. The efficiency of CD8^+^ T cell depletion in the whole blood during the progression of EAE.** The EAE mice received vehicle or 30 mg/kg BrQ treatment starting from day3 post-immunization, while simultaneously treated with either the isotype control or anti-CD8 via intraperitoneal injections. The percentage of CD4^+^ T cell and CD8^+^ T cell was determined in the whole blood on **(A, B)** day10 and **(C, D)** day15 during the progression of EAE, n=6-8 per group. Representative data of two independent experiments.


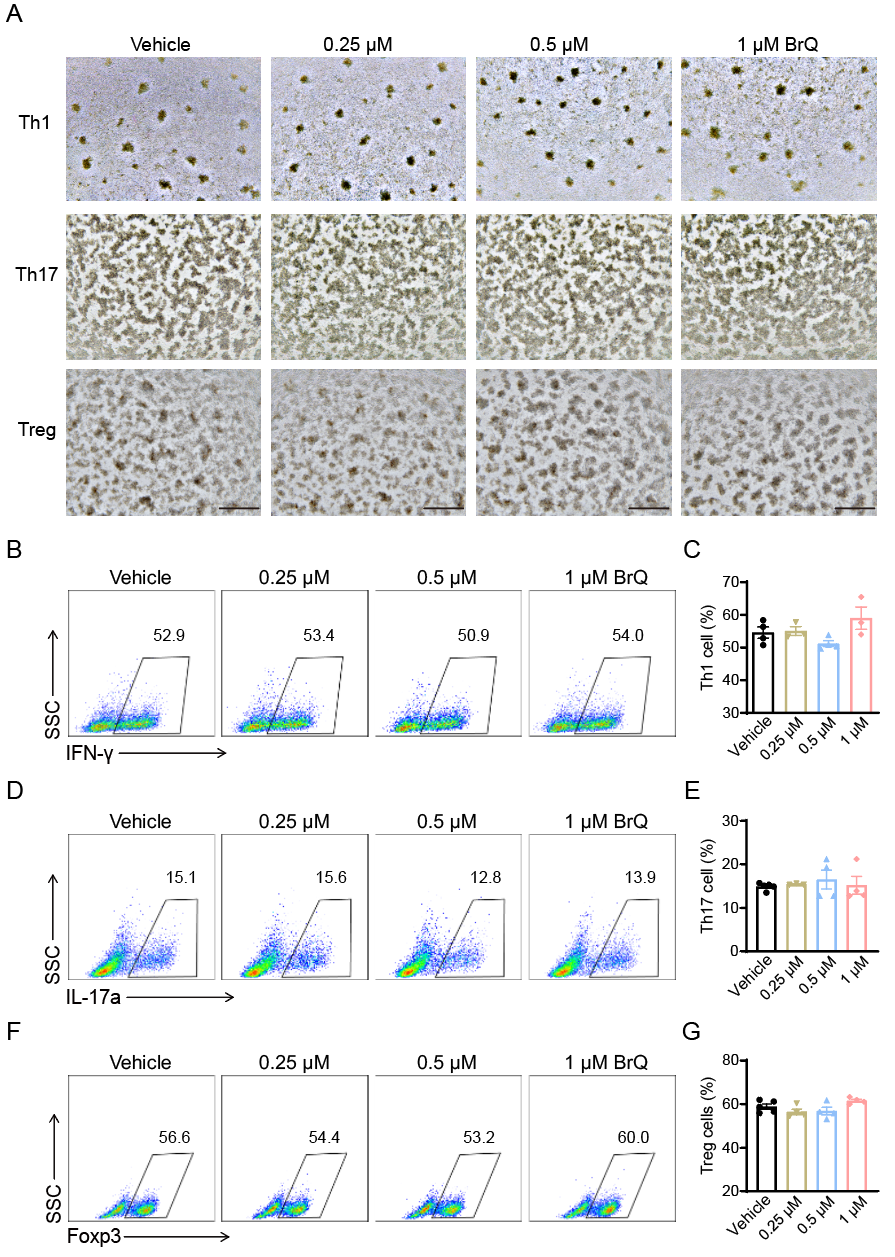


**Fig S4. The Th1, Th17, and Treg cell polarization are not directly affected by BrQ in vitro.** Naive CD4^+^ T cells isolated from the spleen and dLN of 7–8 weeks-old mice were induced to differentiate into Th1 cells, Th17 cells, or Tregs in the presence of various concentrations of BrQ. **A** Clone formation images of Th1 cells, Th17 cells, and Tregs differentiated for 3 days, scale bar, 500μm. **B, D, and F** Representative intracellular staining of IFN-γ, IL-17A, or Foxp3 were analyzed by FACS respectively. **C, E, and G** Statistics of the staining data for IFN-g, IL-17A, or Foxp3. The data are presented as the mean ± SEM (n=3). Representative data of three independent experiments.


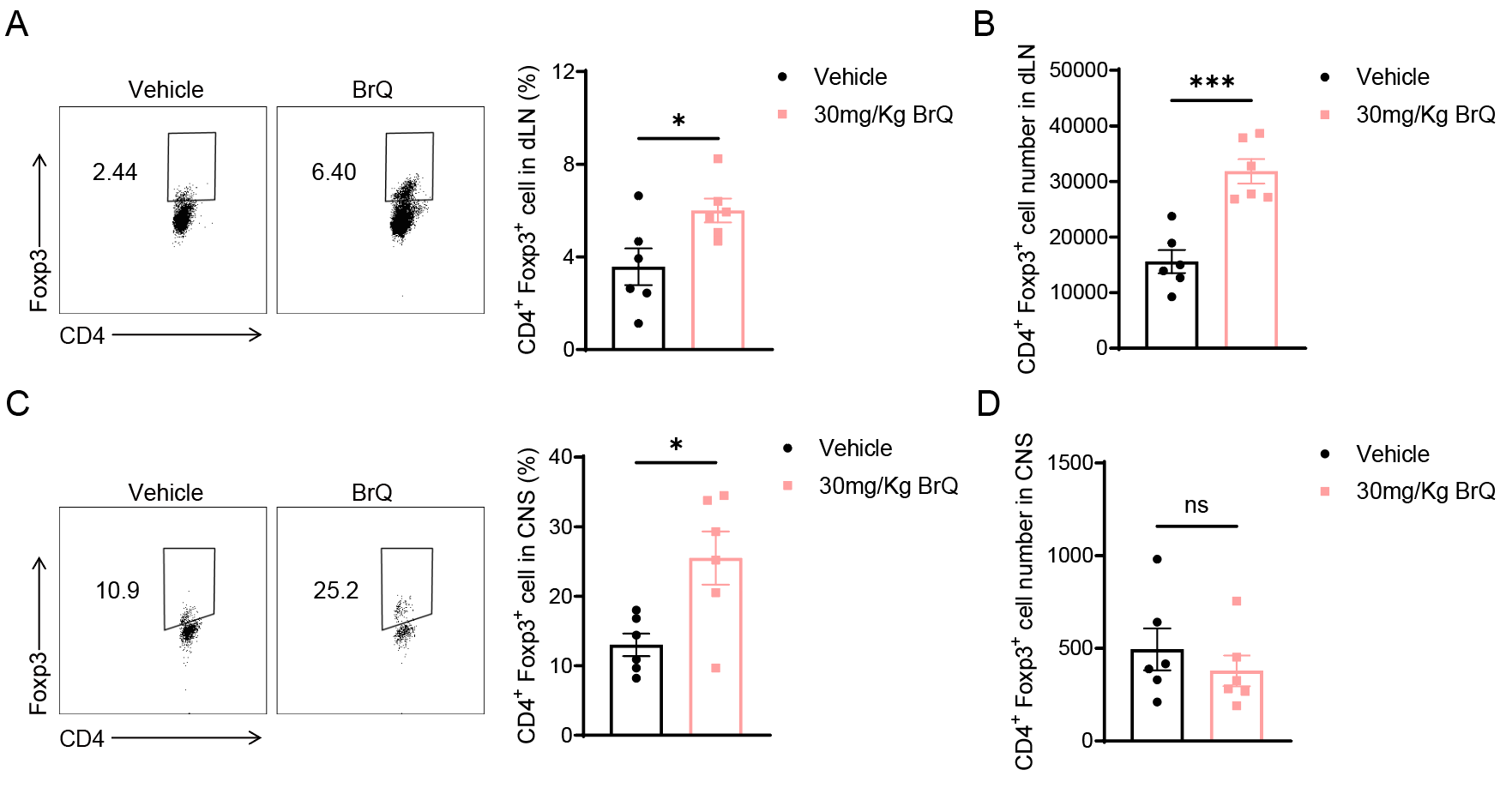


**Fig S5. The effect of BrQ on peripheral dLN and CNS Treg cells in EAE mice. A** Leukocytes were isolated from the dLN of EAE mice treated with 0.5% CMC solution or 30 mg/kg BrQ (oral, starting from day 3) on day 10 post-immunization. Flow cytometric analysis of Treg cells by intracellular staining of Foxp3 in the CD4 gate. **B** Statistical analysis of absolute Treg cell numbers in peripheral dLN (n=6 per group). **C** Total CNS infiltrate lymphocytes were isolated from EAE mice on day 18 post-immunization and Treg cells were quantified by flow cytometry. **D** Statistical analysis of absolute numbers of Treg cells in CNS (n=6 per group). Data represent mean ± SEM. **p* < 0.05, ****p* < 0.001 versus vehicle group (Student’s t test).
